# Dystrophin deregulation is associated with tumor progression in *KIT/PDGFRA* mutant gastrointestinal stromal tumors

**DOI:** 10.1186/2045-3329-4-9

**Published:** 2014-08-09

**Authors:** Maria A Pantaleo, Annalisa Astolfi, Milena Urbini, Fabio Fuligni, Maristella Saponara, Margherita Nannini, Cristian Lolli, Valentina Indio, Donatella Santini, Giorgio Ercolani, Giovanni Brandi, Antonio D Pinna, Guido Biasco

**Affiliations:** 1 Department of Specialized, Experimental and Diagnostic Medicine, Sant’Orsola-Malpighi Hospital, University of Bologna, Bologna, Italy; 2 “Giorgio Prodi” Cancer Research Center, University of Bologna, Bologna, Italy; 3 Pathology Unit, Sant’Orsola-Malpighi Hospital, University of Bologna, Bologna, Italy; 4 Department of General Surgery and Transplantation, Sant’Orsola-Malpighi Hospital, University of Bologna, 40138 Bologna, Italy

**Keywords:** Dystrophin, DMD, Gastrointestinal stromal tumors, KIT/PDGFRA wild type

## Abstract

**Background:**

Intragenic deletions of the dystrophin-encoding and muscular dystrophy-associated *DMD* gene have been recently described in gastrointestinal stromal tumor (GIST), rhabdomyosarcoma (RMS) and leiomyosarcoma (LMS). We evaluated the copy numbers and gene expression levels of *DMD* in our series of GIST patients who were already studied with wide genome assays, to investigate more fully a correlation between dystrophin status and disease annotations.

**Findings:**

Our study highlighted a recurrent intragenic deletion on chromosome X, involving the *DMD* gene that codes for human dystrophin in GIST patients. Of 29 *KIT/PDGFRA* mutant GIST samples, 9 (31%) showed deletions of the *DMD* gene, which were focal and intragenic in 8 cases, and involved loss of an entire chromosome in one case (GIST_188). *DMD* loss was seen in only 5 patients with metastasis, whereas 18 out of 20 patients with localized disease had wild-type *DMD* (*P* = 0.0004, Fisher exact test). None of the 6 *KIT/PDGFRA* WT GIST showed *DMD* alterations.

**Conclusions:**

Our study confirms the presence of *DMD* deletions only in *KIT/PDGFRA* mutant GIST and this event is almost associated with metastatic disease. These findings are, of course, quite preliminary but support development of potential therapeutic strategies that target and restore DMD function in the treatment of metastatic GIST.

## Findings

Intragenic deletions of the dystrophin-encoding and muscular dystrophy-associated *DMD* gene have been recently described in many common human mesenchymal tumors, including gastrointestinal stromal tumor (GIST), rhabdomyosarcoma (RMS) and leiomyosarcoma (LMS) which show myogenic differentiation [1]. In particular, *DMD* deletions were found in 19 of 29 GIST tumors, 3 of 4 RMS tumors and 3 of 7 LMS tumors. Moreover, *DMD* deletions and their protein expression were not found in non-myogenic cancers, or in benign counterparts of GIST, RMS and LMS. In GIST, dystrophin is downregulated in metastatic GIST and primary high-risk GIST, but not in low-risk GIST, which implies this downregulation is a late event in progression of this disease. Finally, dystrophin inhibits myogenic sarcoma cell migration, invasion, anchorage independence and invadopodia formation; and when deregulated, dystrophin restoration inhibits invasiveness and migration in sarcoma cell lines. These data validate dystrophin as a tumor suppressor and likely anti-metastatic factor. In light of these findings, we evaluated *DMD* copy number and gene expression levels in our series of GIST patients who had already been studied with wide genome assays, to investigate more fully the correlation between dystrophin status and disease annotations.

### Patient selection and tumor sample collection

Dystrophin status was evaluated using already-available data from wide genome assays done on tumor specimens collected during surgery and immediately frozen. We included samples from 29 patients with mutant *KIT/PDGFRA* GIST and 6 with wild-type (WT) *KIT/PDGFRA* GIST. Among the *KIT/PDGFRA* WT GIST group, 4 cases were SDH deficient and 2 cases were quadruple *KIT/PDGFRA/SDH/BRAF-KRAS-NF1* WT. Out of all 35 patients, 19 had already been reported [2]. Table 1 shows patients’ clinical and molecular data. The genomic analysis study was approved by local Ethical Committee.

**Table 1:** Clinical and molecular data of the patients included in the study

**ID**	**Sex**	**Age**	**DMD**	**Start**	**End**	**Site**	**Disease status**	**KIT/PDGFRA Mutational status**
**GIST_18**	**M**	**NA**	**loss**	**ex2**	**ex7**	**NA**	**NA**	**KIT exon 11 V559G**
**GIST_20**	**M**	**38**	**loss**	**ex2**		**Small intestine**	**Metastatic**	**KIT exon 11 del MYEQW552–557 Z;**
				**ex8**	**ex17**			**KIT exon 18 A829P**
**GIST_22**	**F**	**76**	**loss**	**ex1**	**ex44**	**Stomach**	**NA**	**PDGFRA exon 18 D842V**
**GIST_25**	**M**	**84**	**loss**	**ex1**	**ex17**	**NA**	**NA**	**KIT exon 11 del WKV557–559 F**
**GIST_26**	**M**	**49**	**loss**	**ex1**	**ex11**	**Stomach**	**Metastatic**	**PDGFRA exon 12 V561D**
**GIST_27**	**M**	**52**	**loss**	**ex1**		**NA**	**NA**	**KIT exon 11 del KV558-559 N**
**GIST_131**	**M**	**58**	**loss**	**ex3**	**ex7**	**Small intestine**	**Metastatic**	**KIT exon 11 del V569_Y578**
**GIST_174**	**M**	**59**	**loss**	**ex1**	**ex7**	**Stomach**	**Metastatic**	**KIT exon 11 L576P**
**GIST_188**	**F**	**57**	**loss**	**All chromosome**		**Small intestine**	**Metastatic**	**KIT exon 11 N564_L576del; KIT exon 17 p.N822K**
GIST_02	F	85	wt	-	-	Stomach	Localized	KIT exon 11 V560D
GIST_04	M	79	wt	-	-	Stomach	Localized	KIT exon 9 ins AY502–503
GIST_05	M	68	wt	-	-	Stomach	Localized	PDGFRA exon 12 ins/del SPDGHE566–571RIQ
GIST_08	M	62	wt	-	-	Stomach	Localized	KIT exon 11 V559D
GIST_09	M	54	wt	-	-	Stomach	Localized	KIT exon 11 ins TQLPYDHKWEFP574–585 at P585
GIST_11	M	65	wt	-	-	Stomach	Localized	KIT exon 11 del WK557–558
GIST_12	F	66	wt	-	-	Stomach	Localized	PDGFRA exon 14 K646E
GIST_13	M	46	wt	-	-	Small intestine	Localized	KIT exon 11 V559D
GIST_14	M	56	wt	-	-	Stomach	Metastatic	KIT exon 11 hom. del WK557–558
GIST_15	F	64	wt	-	-	Stomach	Localized	PDGFRA exon 18 del DIMH842-845
GIST_16	F	62	wt	-	-	Stomach	Localized	KIT exon 11 L576P
GIST_121	M	72	wt	-	-	Stomach	Localized	KIT exon 11 V559D
GIST_124	M	72	wt	-	-	Stomach	Localized	KIT exon 11 ins 1765–1766
GIST_125	F	49	wt	-	-	Stomach	Localized	KIT exon 11 W557R
GIST_129	M	60	wt	-	-	Stomach	Localized	KIT exon 11 del/ins Y553–V559L
GIST_130	F	79	wt	-	-	Stomach	Localized	KIT exon 9 ins A502_Y503
GIST_134	F	65	wt	-	-	Stomach	Localized	KIT exon 11 homoz. V559D
GIST_135	F	60	wt	-	-	Stomach	Localized	KIT exon 11 del W557_E561
GIST_136	M	76	wt	-	-	Stomach	Localized	PDGFRA exon 18 D842V
GIST_150	F	56	wt	-	-	Stomach	Metastatic	KIT exon 11 P551_E554del
GIST_07	F	27	wt	-	-	Stomach	Metastatic	KIT/PDGFRA wild-type (SDH deficient)
GIST_10	M	29	wt	-	-	Stomach	Metastatic	KIT/PDGFRA wild-type (SDH deficient)
GIST_21	F	25	wt	-	-	Stomach	NA	KIT/PDGFRA wild-type (SDH deficient)
GIST_24	F	18	wt	-	-	Stomach	Metastatic	KIT/PDGFRA wild-type (SDH deficient)
GIST_127	F	63	wt	-	-	Ileum	Localized	Quadruple wild-type (KIT/PDGFRA/SDH/BRAF-KRAS-NF1 wild-type).
GIST_133	M	57	wt	-	-	Duodenum	Localized	Quadruple wild-type (KIT/PDGFRA/SDH/BRAF-KRAS-NF1 wild-type).

Patients with DMD loss are shown in bold.

### Copy number analysis

Genomic DNA was extracted with QiaAmp DNA mini kit (Qiagen, Milan, Italy), labelled and hybridized to SNP array Genome Wide SNP 6.0 (Affymetrix) following manufacturer’s instructions. Quality control was performed by Contrast QC and MAPD calculation. Copy number analysis was performed by Genotyping Console and visualized with Chromosome Analysis Suite Software (Affymetrix). Hidden Markov Model algorithm was used to detect amplified and deleted segments.

### RNA sequencing

Whole-transcriptome sequencing was performed on RNA isolated from fresh-frozen tumor tissue with the RNeasy spin-column method (Qiagen). Whole-transcriptome RNA libraries were prepared in accordance with Illumina’s TruSeq RNA Sample Prep v2 protocol (Illumina, San Diego, California). Paired-end libraries were sequenced at 2 × 75bp read length using Illumina Sequencing by synthesis (SBS) technology. Averages of 85 million reads per sample were analyzed. Mapping on the human reference genome was done with TopHat/BowTie software, while expression level of the *DMD* gene was expressed as number of mapped reads.

## Results and discussion

**Figure 1:**
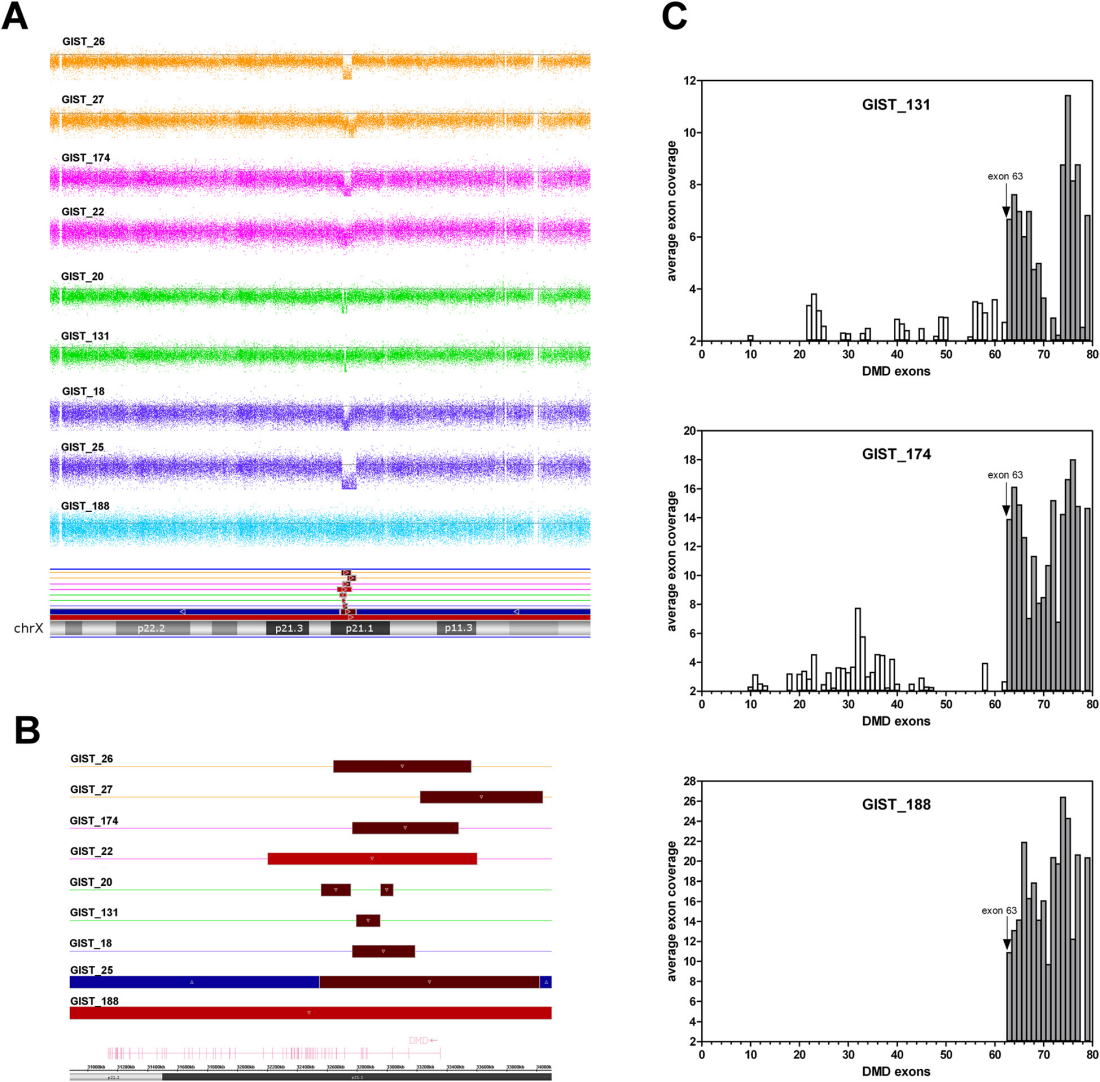
***DMD *deletions and gene expression in KIT/PDGFRA mutant IST samples. (A)** Signal Log^2^ Ratio of copy number data from SNP 6.0 arrays on the X chromosome, showing focal losses and deletion of 1 entire chromosome arm. **(B)** Graphical representation of sizes of the genomic losses of *DMD* gene, located on the reverse X chromosome strand. **(C)** DMD expression showed as average number of mapped reads from RNA sequencing of the samples that carry *DMD* deletions. All cases retain the expression of the short isoform starting from exon 63.

The genome-wide analysis of our series highlighted a recurrent intragenic deletion on chromosome X for the *DMD* gene, which codes for human dystrophin. Nine of the 29 *KIT/PDGFRA* mutant GIST (31%) showed *DMD* gene deletions (Figure 1A), which were focal and intragenic in 8 cases, and involved loss of a whole chromosome in one case (GIST_188; Figure 1B). None of the 6 *KIT/PDGFRA* WT GIST samples had *DMD* alterations.

As *DMD* is an X-linked gene, deletions were nullizygous in males and heterozygous in females. All focal events involved the 5′ portion of the gene, with the region between exon 2 and exon 7 as the most recurrently involved, and an average deletion size of 770 Mb.

RNA-sequencing performed on 3 out of 9 cases with deletions showed that the genomic losses abrogated expression of the largest *DMD* transcript, while preserving expression of the short isoform, with a transcription start site at exon 63 (Figure 1C).

Patients with *KIT/PDGFRA* mutant GIST tended to have higher frequency of *DMD* deletions in more advanced cases, as DMD loss was seen only in the 5 patients with metastasis, whereas 18 out of the 20 patients with localized disease wild-type *DMD* (Table 1; *P* = 0.0004, Fisher exact test).

Our study confirms the presence of *DMD* deletions in *KIT/PDGFRA* mutant GIST. In particular we also observed that this molecular event is associated with more advanced clinical disease such as metastatic tumors. Although these findings are quite preliminary they suggest potential therapeutic strategies that target and restore *DMD* function in treating metastatic GIST. Larger studies are necessary to correlate *DMD* status and specific mutations of *KIT/PDGFRA* receptors to explore novel therapies for GIST that present primary resistance or that initially responds to imatinib but later progresses [3]. As is well known, molecular mechanisms of secondary resistance are various and heterogeneous, the most common being the acquisition of secondary *KIT/PDGFRA* mutations or selection of sub-clones with resistant mutations [4]. In our series, of 7 patients who presented primary mutations in *KIT* exon 11, 2 also had secondary mutations, in *KIT* exon 17 or *KIT* exon 18. The 2 patients with mutations in *PDGFRA* had them in exon 18 D842V or exon 12. The small series does not permit any conclusive considerations on the correlation of DMD involvement with *KIT/PDGFRA* kinase genotype. But as the clinical use of sunitinib and regorafenib after imatinib failure does not completely cover the molecular landscape of the progressing GIST, a novel approach that targets dystrophin deregulation may have relevance in GIST treatment. Moreover, compared with *KIT/PDGFRA* mutant GIST, in our series all patients with KIT/PDGFRA WT GIST—including 4 who were SDH deficient and 2 who had quadruple *KIT/PDGFRA/SDH/BRAF-KRAS-NF1* WT did not present dystrophin deregulation, even those with metastasis, which confirms once again that *KIT/PDGFRA* WT GIST should be considered a distinct disease in molecular background and clinical presentation [5].

## Conclusion

In conclusion, deregulation of dystrophin seems to be associated with GIST progression and more data should be accumulated in order to define it as a therapeutic target.

## Abbreviations

DMD: Dystrophin-encoding and muscular dystrophy-associated; GIST: Gastrointestinal stromal tumors; LMS: Leiomyosarcoma; RMS: Rhabdomyosarcoma.

## Competing interests

The authors declare that they have no competing interests.

## Authors’ contributions

PAM conceived of the study, participated in its design and coordination and drafted the manuscript. AA and UM carried out the molecular genetic studies, participated in the sequence alignment and drafted the manuscript. FF and IV participated in the design of the study and performed the statistical analysis. SM, NM, LC and EG participated in the analysis and interpretation of data. SD carried out the molecular genetic studies. BG, PAD and BG revised the study critically for important intellectual content. All authors read and approved the final manuscript.
